# SKILLSETS FOR FUTURE-READY OLDER WORKERS: SKILLS GAPS AND LEARNING BARRIERS IN SINGAPORE

**DOI:** 10.1093/geroni/igad104.1078

**Published:** 2023-12-21

**Authors:** Vered Seidmann, Yuanyuan Cao, Yuezhong Liu, Ringo Ho, Andy Khong W. H., Dion Goh, Yin-Leng Theng

**Affiliations:** Nanyang Technological University, Singapore, Singapore; Nanyang Technological University, Singapore, Singapore; Nanyang Technological University, Singapore, Singapore; Nanyang Technological University, Singapore, Singapore; Nanyang Technological University, Singapore, Singapore; Nanyang Technological University, Singapore, Singapore; Nanyang Technological University, Singapore, Singapore

## Abstract

Population ageing in Singapore is on a rapid rise as approximately 25% of Singaporeans will be aged 65 or over by 2030. The employment rate of older workers aged 55 and above rose from 24.3% in 2014 to 26.4% in 2021 (MOM , 2022). Older workers are at greater risk of skills mismatch. To enhance employability, governments, and international organizations explore appropriate responses to shifting skills, employability and training. This paper presents results of the qualitative phase in a two-year project that aimed to identify emerging skills, skills gaps, and training barriers among older persons in the Singaporean workforce and provide recommendations to policy makers to better support upskilling and reskilling among employees aged 55 and over. The project included 132 participants (29 age 45-54, 53 age < 55, 35 managers and 15 training providers) from three sectors: Retail, Food Services, and Precision Engineering. The qualitative data revealed skills mismatch resulting from the implementation of technological innovation in daily tasks. Learning barriers among older workers included accessibility to training, mindset and attitudes towards reskilling, fear of technology and training applicability. Recommendations to policy makers included practical solutions to enhance accessibility to course lists and career coaches; identify opinion leaders among older workers and company managers; closer collaboration between employers (inhouse training) and training providers for better follow-up and needs assessments; and utilize Andragogy theory for training design.

